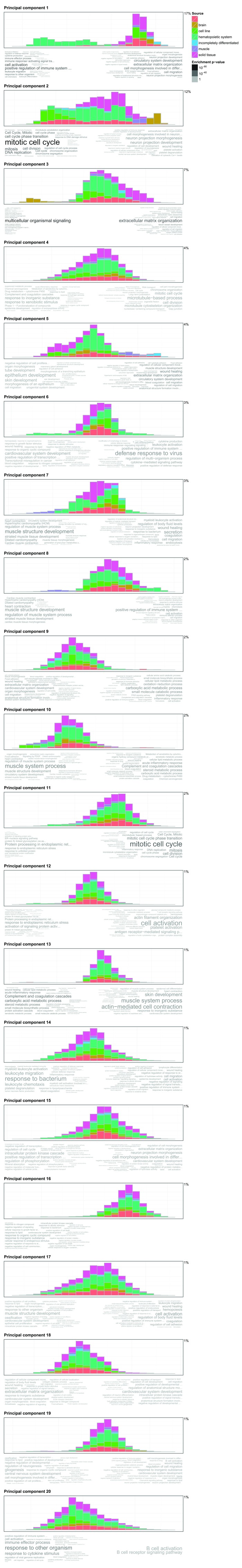# GOsummaries: an R Package for Visual Functional Annotation of Experimental Data

**DOI:** 10.12688/f1000research.6925.1

**Published:** 2015-08-18

**Authors:** Raivo Kolde, Jaak Vilo

**Affiliations:** 1 Institute of Computer Science, University of Tartu, Liivi 2-314, Tartu, 50409, Estonia; 2 Quretec, Tartu, 51003, Estonia; 3 Center for Computational and Integrative Biology, Massachusetts General Hospital, Boston, MA, 02114, USA

**Keywords:** Gene Ontology, word cloud, Gene Set Enrichment analysis, visualisation, Principal Component Analysis, limma

## Abstract

Functional characterisation of gene lists using Gene Ontology (GO) enrichment analysis is a common approach in computational biology, since many analysis methods end up with a list of genes as a result. Often there can be hundreds of functional terms that are significantly associated with a single list of genes and proper interpretation of such results can be a challenging endeavour. There are methods to visualise and aid the interpretation of these results, but most of them are limited to the results associated with one list of genes. However, in practice the number of gene lists can be considerably higher and common tools are not effective in such situations.

We introduce a novel R package, 'GOsummaries' that visualises the GO enrichment results as concise word clouds that can be combined together if the number of gene lists is larger. By also adding the graphs of corresponding raw experimental data, GOsummaries can create informative summary plots for various analyses such as differential expression or clustering. The case studies show that the GOsummaries plots allow rapid functional characterisation of complex sets of gene lists. The GOsummaries approach is particularly effective for Principal Component Analysis (PCA).

By adding functional annotation to the principal components, GOsummaries improves  significantly the interpretability of PCA results. The GOsummaries layout for PCA can be effective even in situations where we cannot directly apply the GO analysis. For example, in case of metabolomics or metagenomics data it is possible to show the features with significant associations to the components instead of GO terms.

The GOsummaries package is available under GPL-2 licence at Bioconductor (http://www.bioconductor.org/packages/release/bioc/html/GOsummaries.html).

## Introduction

As technologies mature, the time and cost of performing microarray and next-generation sequencing experiments is greatly reduced. A wide range of biological questions can be addressed using these experimental approaches. However, several steps of the analysis are often conceptually similar across experiments. At some point of analysis, lists of genes are identified from the data that display interesting behaviour. These lists can represent differentially-expressed genes between two tissues, genes with similar methylation patterns, genes that are close to relevant mutations, etc. Next, these genes are being annotated functionally, by searching for functional terms that are associated with more of them than expected by chance. The latter procedure is called Gene Ontology (GO) enrichment analysis ^1^, and there are many web based tools for this, for example, DAVID ^2^, Babelomics ^3^ and g:Profiler ^4^. The result of GO enrichment analysis is a list of GO terms with associated significance scores. There can be hundreds of significant functional terms associated with one gene list.

Analysis methods often produce many lists of genes instead of only one. For example, clustering analysis can divide genes into tens of clusters, each one of them displaying a distinct biological pattern and potentially unique function. Proper interpretation of the functional analysis results requires that we would also take into account the complex relations between these gene lists. Thus, ideally the underlying experimental data and the functional annotations should be shown together. In practice, the experimental data is usually shown in a single plot while the functional annotations of associated gene lists are given in a series of (long) tables. With this type of representation it is complicated to scan through all the functional terms, while keeping in mind the biological relations between the gene lists and the degree of enrichment of various terms. Therefore, methods that can visually summarise the experimental data and combine it with relevant functional annotations can significantly improve interpretation of analysis results.

For visualising the numeric experimental data there are numerous options, such as heatmaps, barplots, boxplots, etc. However, visualising the GO enrichment analysis results is more complicated, as there are not many options to represent textual data graphically. Many GO visualisation tools aim to reveal the connections between the terms by overlaying them on GO graphs. For example, g:Profiler uses this structure to group the significant results, GOrilla ^5^ overlays the GO graph with enrichment scores, several tools ^6– 10^ visualise the results as a network and REVIGO ^11^ displays significant categories among other options as treemaps and 2D scatterplots. But as the term names would still have to be shown then the resulting plots are physically even larger than the original tables and would not help in comparison of multiple gene lists. To achieve a more compact presentation of results from multiple gene lists, it is possible to display them in a matrix format as a heatmap, where columns represent the lists of genes and rows significant categories.

This is implemented, for example, in the g:Cocoa tool in g:Profiler ^4^ and PloGO ^12^. Although this approach provides a high-level overview of relations between enrichment results, it still tends to create visualisations that are too large to fit a computer screen or a page in print. A promising idea is to represent the enrichment results as word clouds, where the strength of enrichment can be expressed using font size. This is implemented in several tools ^11, 13– 16^, but in all of these cases the emphasis is on the single gene list analysis. One cannot easily combine the results of multiple gene lists or attach the word clouds to the plots of experimental data.

Here we extend the idea of using word clouds to represent GO enrichment results. We implement custom methods to filter GO enrichment results and display them as word clouds. In addition, we define a specific layout to display multiple word clouds, together with the associated experimental data. This allows the creation of concise visual summaries for analyses such as differential expression, clustering or principal component analysis. All the methods are implemented as an R package GOsummaries.

## Methods

### Layout

Examples of the plots produced by GOsummaries can be seen in Figure 2– Figure 4. Although the plots correspond to different data types and analysis methods, the layout stays the same. The plot consists of blocks corresponding to either one or two closely related gene lists, such as a cluster from clustering analysis or up- and down-regulated genes from a differential expression analysis. Each block consists of one or two word clouds representing the GO enrichment results and optionally a panel showing the experimental data related to the lists. The blocks are stacked on top of each other to display multiple gene lists. Depending on configuration one can fit 5–6 blocks on one printed page, however, for exploratory analysis one can easily generate plots with tens of blocks (see Supplementary Figure S2 and Supplementary Figure S3). In this way it is easy to quickly go through and efficiently compare many functional annotations in parallel. As such it does not need to contain all detailed information, but rather aids higher-level understanding. For more detailed analysis users can always refer back to full results from tools like g:Profiler or others.

The content of the panel on top of word cloud(s) is customisable and can display any information that can fit to such space. For example, in case of differential expression and clustering, the panel displays expression of the genes in underlying gene list(s) as boxplots. The y-axis shows the expression level and each boxplot corresponds to one sample. If expression data is not available, then the panel just shows the number of genes.

The word clouds are designed to show the results of a GO enrichment analysis. By default, the GO enrichment analysis is performed by the GOsummaries package itself using the g:Profiler ^4^ web service. However, it is possible to use other type of information, for example, results from other GO enrichment tools or names of the significant genes. The font of the term is sized according to the associated p-values. More specifically, the size of the terms in each cloud is proportional to the - *log*_10_ of the enrichment p-value. As the word placement algorithm tries to use the available space effectively, the term sizes are not comparable between the word clouds. The global strength of enrichment of the terms is color-coded in grayscale.

To make the identification of the lists and their characterisation easier, the content of the gene lists is reflected both in the block title as well as small text next to the panel. For example, in case of differential expression visualisation, the title identifies the groups that were compared and the number of genes that was found is given next to the panel.

### Filtering of GO results

A typical GO-based characterisation of a gene list can contain hundreds of statistically significant GO terms, thus, it is not reasonable to display all of them in one word cloud. As GO defines a hierarchy of biological processes that range from very specific to more general, the GO enrichment analysis results usually contain a number of closely related GO terms. In addition, terms with many associated genes tend to be too general and terms with a small number of genes too specific to give useful information about a larger list of genes. Therefore, it is possible to filter out many terms without losing much information.

There are algorithms such as RedundancyMiner ^17^ that allow to filter the GO enrichment results for redundant terms. However, as g:Profiler, which is used for the functional analysis, has rather good tools for filtering the results, we use those as default.

**Supplementary Figure 1.**
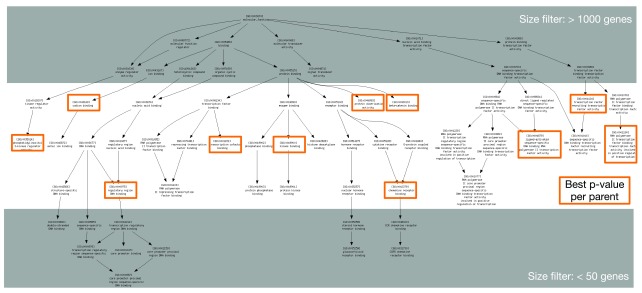


GOsummaries filters the GO terms both based on their size and structural relations, a graphical example can be seen on Supplementary Figure S1. First, it applies the lower and upper limit on the number of genes in the GO terms. By default, it considers GO terms with more than 50 and less than 1000 genes. For removing redundancies GOsummaries uses the hierarchical filtering option of g:Profiler. This divides the results into groups where the terms share parents and takes the one with smallest p-value from every such group. Also by default GOsummaries considers only results from the Biological Process branch of GO and KEGG and Reactome pathway databases. If the number of significant terms is still too high after such filtering, then GOsummaries selects by default 30 terms with the most significant enrichment.

Applying these steps effectively reduces the number of terms while retaining relevant information. The default parameters have proven to be practical for lists of few hundred genes, but all these parameters can be easily changed within the user interface. For example, if one has smaller gene lists, then more specific GO terms can give more appropriate information. Some relevant terms might be lost during the filtering process, but for more specific analysis users can always go back to original results.

### Other Data Sources

Instead of performing the GO enrichment analysis with g:Profiler as described above, a user can supply their own annotations for visualisation as a word cloud. For example, it is also possible for GOsummaries to display results of Gene Set Enrichment Analysis (GSEA) ^18^ or DAVID, or use RedundancyMiner to apply an alternative redundancy reduction step.

The GOsummaries layout can be useful even in cases where we do not use the GO enrichment results. For example, it is natural to show the gene names instead of the GO terms in the word clouds. This option can be useful, for example, for visualising metabolomics or metagenomics data (see Figure 4). It is implemented in several GOsummaries subroutines. For convenience, it can automatically convert various gene identifiers into gene names, using g:Convert web service ^4^.

### Clustering and differential expression

There are several common analysis methods of high-throughput data that create sets of gene lists as a result. For several of such methods we have created specialised routines that extract the gene lists and relevant expression data from the input, run the GO enrichment analysis and display the results. For example, we have created functions that can parse the results from the k-means function for clustering and limma package ^19^ for differential expression.

In both of these cases, the interpretation of the resulting plots is straightforward. The word clouds represent the clusters or significant genes and panels display the expression patterns of these genes.

### Principal Component Analysis

Interestingly, we can apply the GOsummaries approach to Principal Component Analysis (PCA). Usually the results of PCA are depicted as a scatterplot of samples along the first few principal components (PC). The distances between the samples on this low dimensional plot approximate the distance in the actual dataset. Therefore, these plots can reveal outliers and general similarity structure of the samples but very little else.

Actually, PCA reveals much more information than shown on a scatterplot. Each principal component is a weighted sum of original features, such as genes. Thus, the weights, also called loadings, directly show how much influence each feature has to a principal component. In other fields, like psychology, the loadings are routinely used to give an interpretation to the components. However, in bioinformatics this information is often neglected.

In GOsummaries we utilise the information in loadings as follows. First, we take 500 genes with largest positive and negative loadings and run GO enrichment analysis on them. Then we display the results within the GOsummaries layout, where each block represents one principal component. The distribution of samples along the principal component is shown as a stacked histogram, with colour indicating different classes of samples. An example of such visualisation can be seen in Figure 2.

This type of display can be considered complementary to the typical 2D scatterplot representation. If a scatterplot gives an overview of the similarity between the samples, then GOsummaries representation associates a functional interpretation with each of the components. Thus, instead of just observing that a principal component discriminates between two sets of samples, we can also identify the biological processes that underlie this separation. As another advantage, one can display even tens of components in one figure, making it easier to get a comprehensive overview about the PCA results.

### Multidimensional Scaling

For some data types PCA does not work, since the data does not follow its assumptions. Then it is possible to use some other multidimensional scaling (MDS) methods, like principal coordinate analysis. This approach is used, for example, with metagenomics data for visualising similarities in taxon abundances.

In general, the result of a MDS analysis is a matrix with lower dimensionality. As the transformation does not have to be a linear transformation of features, we do not always obtain loadings for the features that could be used for interpreting the new components. Still, we can find correlations between the features and the scaled down components, and perform a statistical test to measure the significance of the correlation. GOsummaries can be applied to the significantly correlated features, much like we use it in case of PCA. It is possible to display either the GO analysis results or the names of significantly correlated features as word clouds.

### Implementation

All the methods are implemented as an add-on package for R statistical computation environment. The GO enrichment analysis is performed through R with gProfileR package that interacts with g:Profiler web toolkit. The figures of experimental data are drawn using ggplot2 ^20^ package.

R was chosen as a platform, thanks to its popularity for genomic analyses. Many of the key statistical algorithms producing the gene lists are specifically implemented in R and, thus, it is natural to integrate the subsequent analyses with it. Unfortunately, this choice constrains the output to static plots, as R does not handle interactivity equally well.

The GOsummaries methodology itself is not restricted to R. For example, we are planning to implement the same approach as a web based tool as well that could take advantage of interactive capabilities of modern Javascript libraries.

### Operation

The package can run on any platform with a relatively recent R installation. When starting from gene lists, k-means clustering or PCA results, the analysis is performed using two steps. First, the GO analysis and filtration is carried out. Then the plot is drawn based on the resulting object. Both steps are automated and usually the analysis can be performed using only two commands. At the same time, all the critical parameters can be customised.

### Preparation of use case data

For comparing the word clouds we used a list of 622 mouse genes. In REVIGO and Cytoscape we used the enrichment results given by g:Profiler. This was the same functional data that was used by GOsummaries. GeneCodis3 and Genes2WordCloud performed the enrichment analysis on their own.

The embryonic stem cell dataset used for clustering was downloaded from ArrayExpress (accession E-TABM-672). We used the processed data matrix and did not apply any additional preprocessing steps. The clustering was performed on 2012 probesets that had standard deviation larger than 1.0.

The gene expression compendium was downloaded from ArrayExpress (E-MTAB-62) as raw data. It was normalised with Robust Multiarray Analysis method ^21^ using default settings.

The example microbiome dataset was provided as an example for the metagenomic biomarker discovery tool LefSe ^22^ and was downloaded from http://huttenhower.sph.harvard.edu/webfm_send/129.

## Results

### Comparison with existing tools

The idea to show GO enrichment results as word clouds is not new and several tools, like REVIGO, Genes2WordCloud, GeneCodis3 and Cytoscape WordCloud already implement it.

However, the usefulness of such an approach depends heavily on the methods used for constructing the word cloud. Most of the published methods follow more or less the approach taken by original word cloud implementation in http://www.wordle.net/, where words are counted and their size reflects their count within the results. However, count of a word within the GO enrichment results is not a good measure of its association with a gene list. With GOsummaries we took a more direct approach, since strength of association is already defined by the enrichment p-value, we just show the full category name scaled according to the p-value.

**Figure 1.**
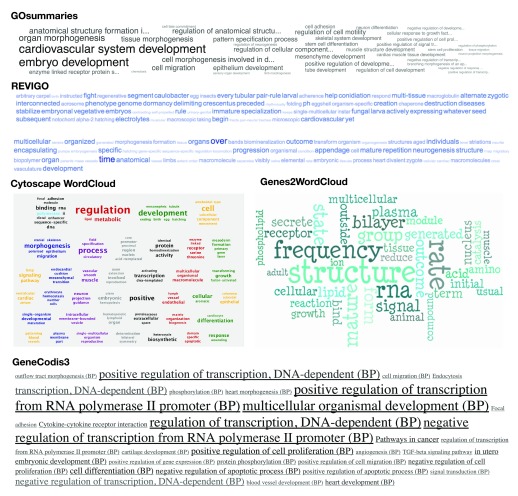
Word clouds describing the same set of genes, created by various tools.

To compare the word clouds produced by different tools, we tried Genes2WordCloud, REVIGO, Cytoscape WordCloud and GOSummaries on a cluster from our embryonic stem cell time series ( Figure 1). The cluster represents genes that are turned on on days 3 and 4 during embryonic development. The GOsummaries word cloud nicely highlights terms that are related to the biological pattern, like “embryo development”, “organ morphogenesis”, “cardiovascular system development”, etc. The results of other word clouds, however, are much poorer. Most of the highlighted words and phrases have nothing to do with the specific expression pattern. GeneCodis3 word cloud emphasises the need for redundancy filtering as most of the largest terms correspond to the same biological process. The word cloud of GOsummaries is also more compact thanks to our custom word placement algorithm that is optimised for fitting longer terms.

On top of these word clouds we have defined a graphical layout that integrates functional annotations of multiple gene lists with experimental data. In summary, GOsummaries produces dense visualisations, summarising large quantities of information, that cannot be recreated easily with existing tools.

## Use cases

### Embryonic stem cell time-series

As a practical example we used data from an experiment, where gene expression was measured in developing embryoid bodies at nine time points starting from stem cells ^23^. The goal of the experiment was to understand temporal patterns of gene regulation that guide the differentiation process.

**Figure 2.**
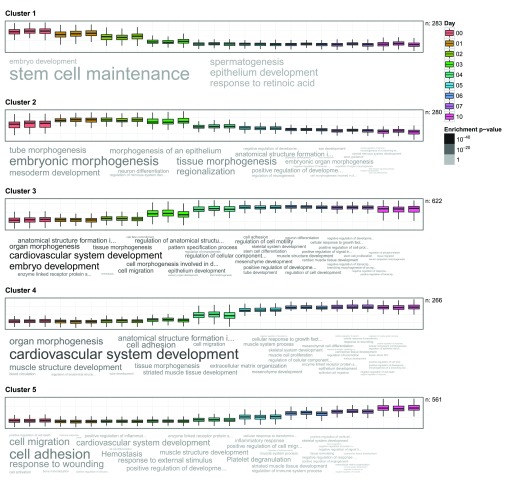
GOsummaries representation of k-means clustering results. Each cluster is described by a boxplot showing expression of the genes and a word cloud showing the most significant GO results. In the boxplot, each box corresponds to one sample and the y-axis shows the expression values. In the word clouds the size of the words is proportional to - *log*_10_ of enrichment p-value within one word cloud. The absolute enrichment strength of terms (words) is color-coded in grayscale.

To achieve these goals, it is natural to first cluster the genes from high-throughput analysis into groups with similar behaviour and then characterise the groups functionally using GO enrichment analysis. GOsummaries visualisation is helpful in the interpretation and presentation of the clustering results. Figure 2 shows the GOsummaries results of k-means clustering ( *k* = 5) on the time series. The main trends in the data are immediately clear from the figure. The genes that are related to stem cell maintenance are gradually turned off in the first few days. At the same time developmental genes are turned on in waves: first the embryonic morphogenesis and mesoderm development genes, then in two waves the cardiovascular system-related genes and, finally, the cell adhesion and immune system related genes.

**Supplementary Figure 2.**
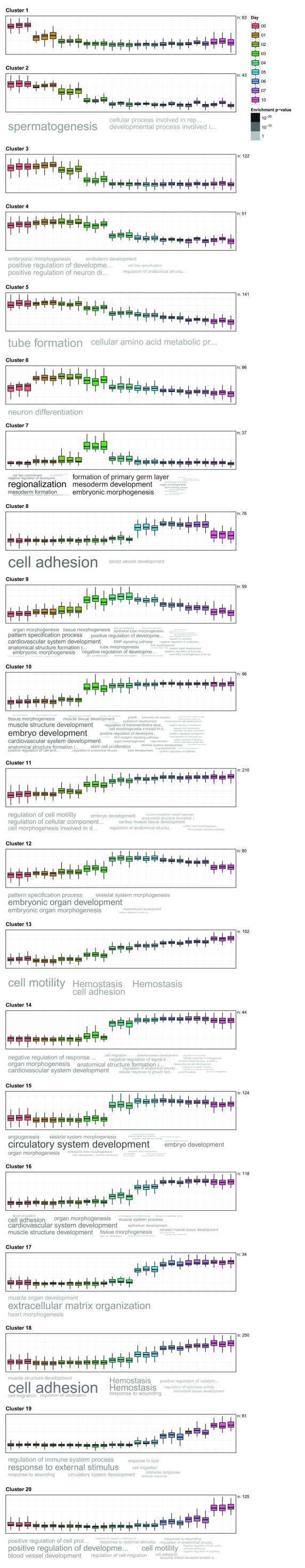


Five clusters presented in Figure 2 may not give an adequate overview of the dataset, increasing the number of clusters can reveal more interesting patterns. Supplementary Figure S2 shows the results for clustering where *k* = 20. Using GOsummaries to display the results makes the comparison of the clusterings easy. It is possible to see what clusters were divided, how did the division influenced the annotations and if any new interesting patterns emerged. For example, Cluster 4 in Figure 2 has been divided into three clusters in Supplementary Figure S2 (clusters 14–16). Although the expression patterns look very similar, the annotations are somewhat different between the new clusters. Cluster 7, that has a very distinct functional profile is a nice example of a new pattern emerged in the second clustering. In some other cases the new clusters have weak annotations, suggesting that they can be either combined together or ignored.

### PCA on the gene expression compendium

To illustrate the utility of the PCA visualisation of GOsummaries we used the gene expression compendium published by Lukk *et al* ^24^. This dataset is a collection of publicly available gene expression data covering 5372 samples from 206 studies, with annotations that were thoroughly re-curated by the authors. The analysis in the original article was based on principal component analysis. They inspected the first four principal components and related them with the cell types and tissues by visual inspection of the distribution of samples.

Using GOsummaries on this data could improve the analysis in two aspects. First, the GO annotations would add another dimension to the interpretation of the principal axis. Second, a dataset that is as diverse as this one may enclose more interesting features beyond the first four principal components; and therefore its analysis could directly benefit from GOsummaries that can easily create plots with tens of principal components to be screened for interesting associations.

**Figure 3.**
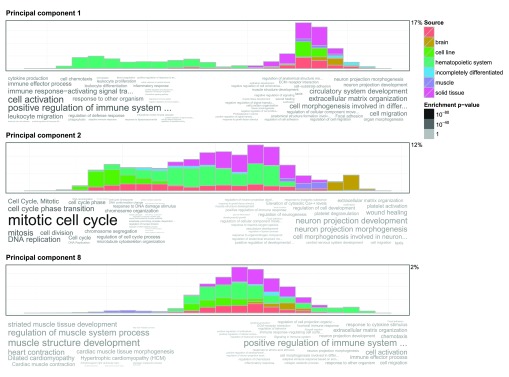
GOsummaries representation of PCA results. Each component is described by a histogram showing the distribution of samples along the principal axis and word clouds showing the GO annotations for most influential genes. The left and right word clouds represent 500 genes with largest negative and positive loadings respectively. The percentages next to histograms show the percentage of variation explained by each component.

We applied the GOsummaries approach and plotted the first 20 principal components ( Supplementary Figure S3). Then we selected 3 additional interesting components for Figure 3. The GO annotations of the first components match well with the names and descriptions of the components presented in the original article. First component was named “Hematopoietic axis”. Fittingly, the GO annotations were strongly related to immune function in the negative end of the axis where the blood cells were clustered. Second component was named “Malignancy axis” and the most dominant GO annotations related to cell line and cancer samples were cell cycle and DNA replication. But there are informative components beyond the first four that were studied in the original article. For example, the eighth component clearly distinguishes muscle cells from everything else and indeed the GO annotations are also muscle related. Several other cases where GO annotations match well with the distributions of samples along different principal axes can be found in the Supplementary Figure S3, where first 20 principal components are shown.

In these examples we already knew what to expect from the GO annotations. In practice, however, there are often situations, where we can identify clear subclasses from the PCA results, but cannot characterise them any further. In these cases, the GO annotations could give invaluable insights to explain the patterns in the data.

### Metagenomic Case Study

Principal Coordinate Analysis (PCoA) is a common approach for visualising taxon abundance data in metagenomic studies. The method is closely related to PCA and its results are usually presented in a similar manner as two- or three-dimensional scatterplots, with the same shortcomings. Thus, using GOsummaries on PCoA results of metagenomics data could make the results more interpretable.

**Figure 4.**
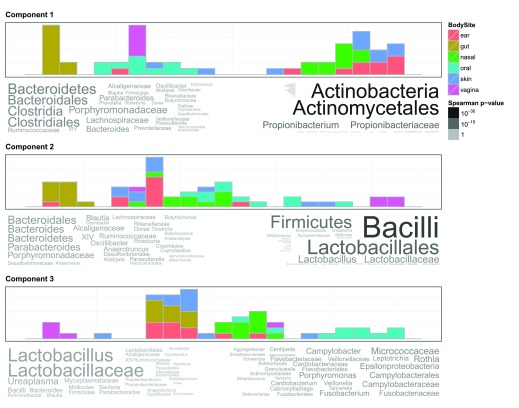
GOsummaries representation of PCoA on metagenomic data. Each component is described by a histogram showing the distribution of samples along the principal coordinate axis and word clouds showing most correlated features. The sizes and colours of the taxons in word clouds are proportional to - *log*_10_ of the Spearman rank correlation test p-value. The right and left word clouds represent taxons that were significantly either correlated or anti-correlated respectively with the principal coordinate.

As an example, we use a small subset of Human Microbiome Project 16S dataset that contains samples from various body sites. We applied PCoA using Bray-Curtis dissimilarity on the data to identify three principal coordinate axes. Then we associated taxons from the original data to the principal coordinates using Spearman correlation test and displayed the results using GOsummaries ( Figure 4).

The traditional scatterplot view would have told us only that there is a clear difference in microbial composition in different body sites. However, the GOsummaries version also identifies the taxons that contribute to the difference. For example, according to the first principal coordinate, the skin, ear and nasal sites have more abundant Actinobacteria, previously shown to be a dominant component of skin microbiota ^25^. According to second and third principal coordinates, vaginal sites tend to have more abundant Lactobacillus, previously shown to be an important part of healthy vaginal microflora ^26^.

GOsummaries visualisation added considerable analysis depth to the PCoA of microbiome data, by revealing underlying differences between experimental groups.

## Summary

Here we describe an R package GOsummaries that can be used for visualising functional annotations. By showing the annotations as word clouds and combining them with plots of underlying experimental data it is possible to create concise summaries of common analyses. The approach can be applied to any gene list, but is especially useful for clustering, PCA and differential expression results. We show the utility and wide applicability of the tool through three case studies. In comparison with other tools, we demonstrate that GOsummaries word clouds are compact but still manage to convey most relevant biological information. As the analysis pipeline used by GOSummaries is highly automated, it is easy to use and can be useful in many practical situations.

## Software availability

### Software access

http://www.bioconductor.org/packages/release/bioc/html/GOsummaries.html

### Updated source code

https://github.com/raivokolde/GOsummaries

### Source code as at time of publication

https://github.com/F1000Research/GOsummaries

### Archived source code as at time of publication

( *F1000Research* TO GENERATE)

### Software License

GPL-2

## Figures and Tables

**Supplementary Figure 3.**