# Supplementary material for: GOsummaries: an R Package for Visual Functional Annotation of Experimental Data
Source: F1000Res. 2015 Aug 18;4:574. [Version 1] doi: 10.12688/f1000research.6925.1 (PMC4743157; doi:10.12688/f1000research.6925.1)
Supplement: Peer review history

# Peer review: Referee response for version 1

**DOI:** 10.5256/f1000research.7457.r10691

**Published:** 2015-10-14

**Authors:** Hilary Coller

**Affiliations:** 1 Department of Molecular, Cell and Developmental Biology, University of California Los Angeles, Los Angeles, CA, USA

The authors have developed a method for summarizing information Gene Ontology searches in a format that makes it easier to understand. They focus on the most relevant Gene Ontology terms and make a word picture with the size of the words indicating their strength in the Gene Ontology results. There is also a method to take principal components of a gene expression dataset and visualize the behavior of different components in each sample along with the word pictures describing the gene ontology categories. The formatting is easy to understand and I expect that this software will be valuable for scientists investigating gene expression analysis.

I have read this submission. I believe that I have an appropriate level of expertise to confirm that it is of an acceptable scientific standard.

# Peer review: Referee response for version 1

**DOI:** 10.5256/f1000research.7457.r10188

**Published:** 2015-09-08

**Authors:** Shaillay Dogra

**Affiliations:** 1 Vishuo BioMedical Pte Ltd, Singapore, Singapore

GOsummaries is another nice way to summarize visually biologic process related data. Novel tools to visualize information in innovative and meaningful ways are always a welcome addition. The application on top of data from PCA is interesting. So is the informative use on microbiome metagenomics data. Overall, GOSummaries sounds like a good package on top of other useful packages plus novel functions of its own merit. The manuscript is very well-written, is easy to read and follow.

Specifically:

1. Is it possible to add functionality to keep term sizes across different word clouds to a fixed-scale option that then enables a user to be able to compare across separately generated figures (I note that authors have already mentioned this is not possible currently and also the reasons thereof).
2. In Figure 1, authors have generated word-clouds on same input data using different available software options including their own GOsummaries. For the benefit of the readers, could the authors discuss on why different tools emphasize different words in their vizualitaions? What underlying assumptions of the different tools make the same input list be represented in different word sizes? For ex. GOsummaries emphasized: cardio vascular development; Cytoscape emphasized: regulation; genes2wordcloud emphasized: frequency, structure --- why is this so? For a general reader more on experimental biology background this may seem confusing and as if the tools are unreliable. Some discussion from authors on this aspect will be beneficial to the readers.

I have read this submission. I believe that I have an appropriate level of expertise to confirm that it is of an acceptable scientific standard.
